# Concurrent Spinal and Intracranial Subdural Hematomas as a Cause of Near-Fatal Low Back Pain in the Chiropractic Office: A Case Report

**DOI:** 10.7759/cureus.31900

**Published:** 2022-11-26

**Authors:** Eric C Chu, Robert J Trager, Yuan S Nga, John S Shum

**Affiliations:** 1 New York Chiropractic and Physiotherapy Centre, EC Healthcare, Kowloon, HKG; 2 Chiropractic, Connor Whole Health, University Hospitals Cleveland Medical Center, Cleveland, USA; 3 Chiropractic, Logan University, Chesterfield, USA; 4 Radiology, Hong Kong Advanced Imaging, EC Healthcare, Kowloon, HKG

**Keywords:** trauma, subdural hematoma, elderly, low back pain, chiropractic

## Abstract

In older individuals, minor trauma may cause potentially fatal intracranial subdural hematoma (SDH). Rarely, these patients present with only low back and radicular pain as gravity redistributes the SDH to the lumbar spine.

A 69-year-old male presented to a chiropractor with a 10-day history of acute on chronic low back pain, which radiated into his lower extremities bilaterally, involving weakness and difficulty walking, and a ground-level fall onto his elbows 16 days prior. He had visited his primary care provider, orthopedist, and traditional Chinese medicine practitioner, received oral analgesics and three ketorolac injections, and had lumbar radiographs, followed by acupuncture, cupping, and spinal manipulation without lasting relief. Considering the patient’s concerning presentation, the chiropractor ordered lumbar magnetic resonance imaging (MRI) on the first visit, revealing findings suggestive of late subacute lumbar SDH, and recommended urgent brain MRI and neurosurgical referral. The patient went to an orthopedic surgeon at a nearby hospital, becoming disoriented upon presentation, prompting admission. Brain MRI confirmed bilateral chronic intracranial SDH, prompting emergency hematoma evacuation via burr hole craniostomy. The patient’s gait rapidly improved, and the pain subsided over the following two weeks.

This case highlights an older male identified as having spinal SDH by a chiropractor, leading to referral and surgery for concurrent life-threatening intracranial SDH. Clinicians should be aware that spinal SDH may stem from asymptomatic intracranial SDH and should be suspicious of SDH in older individuals after a fall, signs of which warrant emergency referral for MRI and surgical evaluation.

## Introduction

Subdural hematoma (SDH) is a type of hemorrhage in which blood accumulates in the subdural meningeal space. Intracranial SDH is relatively common and represents the leading cause of death related to ground-level falls in individuals aged 65 years and older [1]. In comparison, spinal SDH is rare, making up only 4% of cases of spinal hematoma [2]. Even more rarely, an intracranial SDH may migrate downward, causing an additional lumbar SDH [3,4], which may lead to a confusing clinical picture with low back and radicular pain without cranial symptoms [3,5].

Concomitant intracranial and spinal SDH is usually the result of trauma [6]. While high-energy trauma is typically required to cause SDH in younger individuals, in older individuals, SDH may result from minor trauma [7]. In such patients, any type of acceleration/deceleration injury may cause SDH [8], with up to half of older patients not having any direct head trauma [9]. Aside from trauma and older age, risk factors for SDH include coagulopathy, anticoagulant and antiplatelet medications, and alcoholism [9,10].

The subdural space is a potential space between the closely apposed layers of dura and arachnoid mater [11]. In elderly individuals, bridging veins in this space are more susceptible to tearing as age-related brain atrophy allows for greater brain movement and stretching of these veins [9]. Conversely, the spinal subdural space contains few blood vessels and is less often a source of bleeding [3]. However, as the intracranial and spinal subdural spaces are continuous with one another, intracranial SDH may redistribute to the lumbar subdural space as a result of gravity [5,6]. A recent review in 2020 identified 16 cases of spinal SDH that occurred in relation to intracranial SDH that had migrated inferiorly [3].

Chiropractors are portal-of-entry healthcare providers that often manage spinal disorders such as low back pain using spinal manipulation [12], a form of manual therapy directed to the joints of the spine [13]. While these providers may only rarely encounter serious pathology such as SDH [14], this condition is important to identify as it is potentially fatal and represents a contraindication to spinal manipulation [15]. According to a literature search of PubMed, the Index to Chiropractic Literature, Google Scholar, and a recent review paper [16], on November 17, 2022, we identified only a single case of SDH identified by a chiropractor [17].

Considering that concurrent intracranial and spinal SDH is an extremely rare but potentially fatal condition that may present to providers that manage spinal conditions, we report a patient with lumbar SDH who presented to a chiropractic office. The patient was ultimately diagnosed with intracranial SDH and responded positively to brain surgery.

## Case presentation

A 69-year-old Asian male with a medical history of controlled hypertension and diabetes mellitus for the previous 10 years presented to a chiropractor with a 10-day history of an exacerbation of chronic low back pain that began acutely six days after a ground-level fall in which the patient tripped and fell forward, landing on his right elbow and wrist. The patient noted that he did not hit his head. While the patient previously had mild localized low back pain before and immediately after the fall, six days after the fall, he developed frequent low back pain rated 10 out of 10 in severity with radiation into the bilateral gluteal region, posterior thighs, and legs. He noted weakness in his lower extremities bilaterally when walking and when transitioning from a seated position to standing. Symptoms were aggravated lying supine and worse at night. He noted nocturnal leg cramping, which awakened him from sleep, and severe low back pain upon waking. He also noted that bending forward, prolonged sitting, and transitional movements aggravated his pain. He denied any sensations of numbness in the lower extremities, bowel or bladder impairments, headache, or dizziness, and he had no history of falls preceding his recent fall.

The patient took an ace inhibitor, metformin, and aspirin (81 mg per os) for the previous 10 years. He was a retired policeman, and until his recent fall, he was active with exercise. He was a current smoker and social drinker. He had no family history of cancer or spinal disorders. His World Health Organization Quality of Life score was 52%.

**Figure 1:**
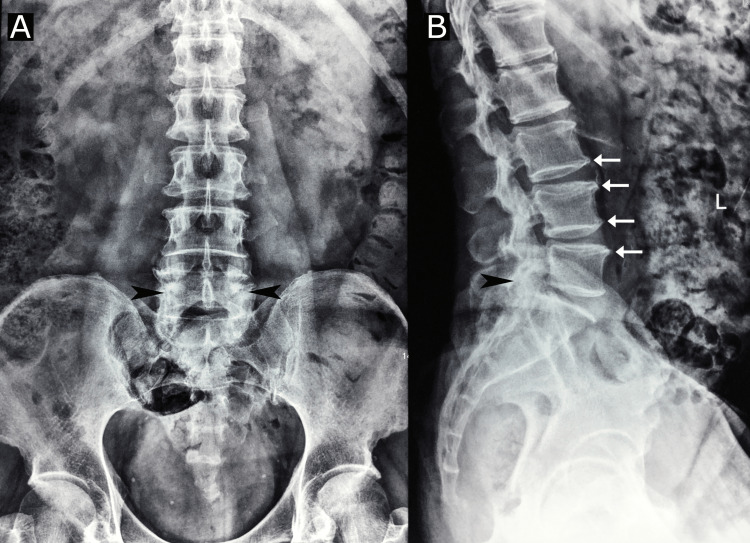
Lumbar spine radiographs Anteroposterior (A) and lateral (B) radiographs taken prior to presentation to the chiropractor reveal facet sclerosis at L5/S1 (arrowheads), while the lateral radiographs (B) reveal anterior vertebral osteophytes (arrows) and marked reduction in lumbar lordosis. These findings are consistent with mild degenerative spondylosis of the lumbar spine. There are no signs of serious pathology.

After the fall, the patient initially only had elbow pain and swelling and saw a traditional Chinese medicine provider for this complaint, which resolved within a couple of days. After his low back pain became aggravated, he presented to his primary care provider who prescribed tramadol and acetaminophen per os and performed a gluteal injection of ketorolac. The provider referred the patient to the emergency department; however, the patient declined and was instead referred to an orthopedist. The orthopedist ordered lumbar spine radiographs, which revealed mild lumbar spondylosis (Figure 1). Considering oral medications had not significantly alleviated his pain, the orthopedist performed another ketorolac injection in the gluteal region, which reduced the patient’s low back pain for one day. This procedure was repeated later that week with the same result.

Preceding his chiropractic visit, the patient again saw a traditional Chinese medicine provider who performed acupuncture, cupping therapy, and lumbar spinal manipulation, which afforded the patient only transient relief from his low back pain. The patient sought a chiropractor for another opinion given his severe pain and limited response to other therapies.

Upon examination by the chiropractor, the patient was observed to have a slow gait and limp in which he struggled to flex his hips to lift his knees when walking. However, he was able to heel walk and toe walk without difficulty and balance in a single-leg stance without help. His active lumbar range of motion was severely limited in all planes. Straight leg and well leg raising reproduced his lower extremity pain each at 30 degrees. Likewise, the slump test was markedly painful bilaterally, such that the patient could not fully straighten his knees in this position. Combined flexion, abduction, and external rotation of the hip and lumbar extension quadrant tests were also both painful, while prone knee bending was painless. A cranial nerve examination and muscle strength, reflex, and sensation tests were normal. Spinal palpation revealed motion restriction with tenderness at L3/4 and L4/5, and hypertonicity and tenderness of the quadratus lumborum and gluteal muscles bilaterally.

**Figure 2:**
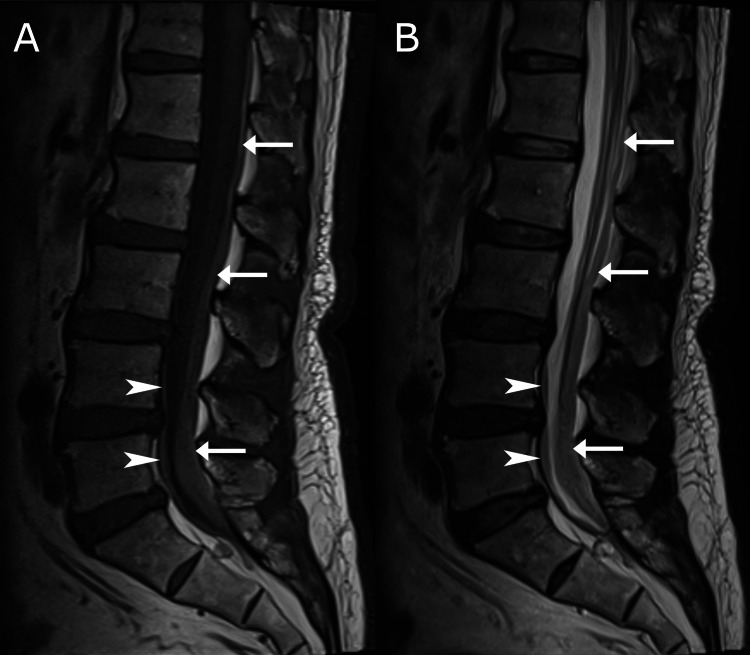
Sagittal lumbar MRI A T1-weighted (A) and T2-weighted (B) mildly hyperintense signal layer measuring up to 1 cm in anteroposterior diameter is seen along the anterior and posterior aspect of the spinal canal from T12/L1 to S1/2 levels, with tapered superior and inferior edges, appearing intradural in location (arrows), consistent with subdural hematoma. A thinner layer with a similar signal is seen at the anterior aspect of the spinal canal from L4 to S1/2 levels, measuring up to 0.4 cm in the anteroposterior diameter, also appearing intradural (arrowheads). MRI: magnetic resonance imaging

**Figure 3:**
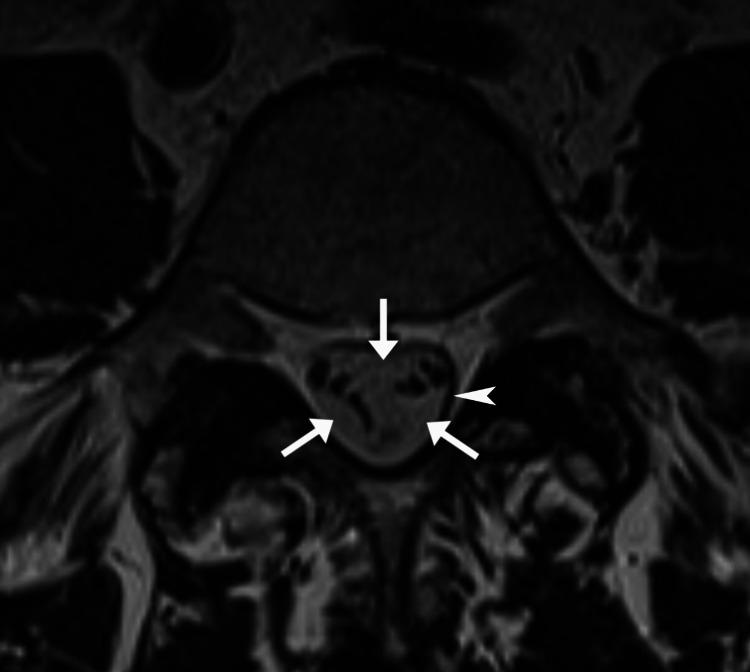
Axial lumbar MRI A T2-weighted section at the level of the L5 vertebra shows crowding of nerve roots centrally with denticulate ligaments and midline dorsal septum separating the subdural collections into an anterior collection and two posterolateral collections (arrows). The hematoma also follows the rounded contour of the thecal sac (arrowhead), a feature that helps distinguish this condition from an epidural hematoma. MRI: magnetic resonance imaging

Given the patient’s older age, recent fall, and lack of improvement with previous therapies, the chiropractor considered an occult lumbar vertebral compression fracture as the working diagnosis and ordered lumbar spine magnetic resonance imaging (MRI) and dual-energy X-ray absorptiometry (DXA) at the first visit, which were performed two days later. While the DXA findings were normal, MRI revealed findings consistent with SDH causing crowding of the cauda equina (Figure 2 and Figure 3). The suspected SDH was mildly hyperintense on T1-weighted and T2-weighted sequences, findings that were consistent with a late subacute hematoma [11,18]. These findings were compatible with a fall and subsequent development of SDH over the previous 16 days.

The chiropractor consulted with the radiologist and oncologist in the affiliated clinic system. Given the findings that were concerning for SDH and considering the patient’s age, use of aspirin, and recent fall, the specialists advised the chiropractor that the patient required immediate brain MRI, given there could be an intracranial source of bleeding, and recommended emergency referral to a neurosurgeon. Accordingly, the chiropractor informed the patient of the seriousness and likelihood of SDH and recommended a brain MRI and neurosurgical referral. However, the patient preferred to visit an outside orthopedic surgeon and saw this provider two days later.

Upon evaluation by the surgeon, the patient began to demonstrate signs of disorientation in addition to his low back pain and gait disturbance. Given the patient’s new symptoms and history of a fall, the surgeon admitted the patient to the hospital and ordered a brain MRI to evaluate for concurrent intracranial hemorrhage. This MRI revealed findings suggestive of chronic SDH at the cerebral convexes bilaterally, measuring 1.5 cm on the right and 1.3 cm on the left. There was a mass effect with compression of the cerebral hemispheres bilaterally and a midline shift toward the right side. These imaging findings were consistent with the development of an SDH over the past 18 days following the patient’s fall.

A neurosurgeon performed a burr hole craniostomy the following day, allowing evacuation of the SDH. Two days later, the patient’s disorientation and ability to walk had improved, and he was discharged to recover at home. Over the subsequent two weeks, his low back pain progressively improved. The patient provided written consent for the publication of this case and its accompanying images.

## Discussion

This case represents one of the first descriptions of SDH identified by a chiropractor in an older man who developed severe low back pain with a delayed onset after a fall. This case presented a challenge, with SDH going unrecognized for over two weeks. Fortunately, after presenting to a chiropractor who identified the lumbar SDH via MRI, the patient was referred to a surgeon, who ultimately identified and surgically evacuated the intracranial SDH.

We suspect that the source of the bleeding was intracranial in this case and that blood migrated to the lumbar region rather than there being two distinct sites of bleeding. Our conclusions are consistent with prior research on concurrent intracranial and spinal SDH [3,5] and supported by the high prevalence of intracranial SDH in elderly individuals after falls [1]. There was also no acute intrinsic lumbar spine pathology evident on MRI (i.e., fracture) that would otherwise explain the patient’s gait abnormality or the lumbar SDH. Finally, the patient’s low back pain was not severe until six days after the fall, which is consistent with a previous review that reported a symptom delay of 12.5 ± 15.1 days between trauma and the onset of symptoms related to spinal SDH [3]. Accordingly, we suspect that the patient’s spinal SDH developed progressively after the fall until becoming symptomatic.

We suggest that the patient’s gait difficulty was mostly linked to his intracranial rather than spinal SDH and thus represented a false localizing sign. The patient’s gait could best be described as gait apraxia, which is a functional gait abnormality involving weakness only when walking and no cerebellar dysfunction or sensory loss [19]. The current patient matches this description as he was neurologically intact and did not display balance abnormalities yet had an unusual, slow gait with apparent difficulty initiating hip flexion. Also supporting this hypothesis was the rapid improvement of his gait following brain surgery, as the lumbar SDH was treated conservatively and was left to resolve on its own. Gait apraxia has been reported previously among patients with chronic intracranial SDH [20] and is suspected of localizing to lesions of the supplementary motor area of the brain [21].

Aside from older age and trauma, the patient had risk factors for bleeding. Aspirin, which acts as an antiplatelet agent, increases the risk of SDH even at a low dose (risk ratio: 1.5) [22]. Further, the patient received three ketorolac injections after his fall, which potentially could have exacerbated the SDH. Ketorolac, which inhibits prostaglandins and subsequently platelet formation, significantly prolongs bleeding time and is contraindicated in patients with hemorrhage, including cerebrovascular bleeding [23,24].

Although acupuncture and spinal manipulation have been rarely reported to cause spinal hematoma [25], we suggest that in the current case, these therapies, which were applied two weeks after the fall, were applied when the hematoma was likely already present per its MRI characteristics. While it was fortunate that these therapies did not exacerbate the patient’s symptoms or appear to cause the lumbar SDH, spinal manipulation remains a contraindication in SDH [15]. Instead, the priority for providers of spinal manipulation should be the referral of such patients for surgical evaluation.

Clinicians should be vigilant to detect any SDH in older patients who have fallen and should perform a thorough examination, including a review of medications, gait analysis, and neurological examination [3]. MRI is the optimal imaging modality for those suspected of spinal SDH [11,25], while either computed tomography or MRI is appropriate for suspected intracranial SDH [22]. As illustrated in the current case, the presence of spinal SDH or any signs of elevated intracranial pressure such as disorientation, headache, nausea, or vomiting warrants brain neuroimaging to rule out intracranial SDH, regardless of a history of direct head injury [6,26]. The detection of SDH warrants an emergency surgical referral.

Due to the limited number of cases, the optimal management for concurrent intracranial and spinal SDH is not clear. However, among previously reported similar cases, intracranial SDH was preferentially treated surgically, while lumbar SDH was observed [6,26], as in the current case. While craniotomy and burr hole craniostomy have both been used for intracranial SDH, a recent systematic review reported that the less invasive burr hole approach, as used in the current case, is associated with a reduced recurrence and reoperation rate and shorter duration of operation compared to minicraniotomy [27].

Chiropractors not only treat low back pain but have an important role in its diagnosis [28]. These providers should screen for red flags for serious pathology [28], such as those that were present in the current case, including recent trauma, older age, and night pain [29]. While SDH may be challenging to identify from the history and examination alone, such combinations of red flags typically prompt MRI [30]. Regardless, if SDH is suspected, spinal manipulation should be withheld until MRI can be performed [15].

There are several limitations in the current case. First, although the clinical and imaging features and response to surgery suggested an intracranial source of SDH, two separate sites of bleeding (e.g., intracranial and spinal) cannot be completely ruled out. Second, detailed surgical records and brain MRI were unavailable from the outside hospital, and this information was limited to the records and MRI report that the patient sent. Third, the chiropractic scope of practice differs in each country, and providers unable to order MRI could instead directly refer patients with complicated low back pain and red flags. Fourth, it is possible that subtle signs of elevated intracranial pressure or upper motor neuron lesion were missed by the examining providers before the patient saw the neurosurgeon, as the clinical focus appeared to be more on the lower back pain.

## Conclusions

This case describes an older male presenting with low back pain and gait abnormality with delayed onset after a fall, ultimately identified as concurrent spinal and intracranial SDH via MRI. This case highlights the important clinical lessons that SDH should be considered in older adults following even minor trauma and that spinal SDH may result from asymptomatic intracranial SDH. When imaging or clinical features are suggestive of spinal and/or intracranial SDH, an emergency surgical referral should be made considering SDH is potentially fatal.
